# Systematization of Steps for Printing 3D Models of Orthopedic Deformities

**DOI:** 10.1055/s-0042-1748816

**Published:** 2022-06-06

**Authors:** Mariana Demétrio de Sousa Pontes, Carlos Henrique Ramos, Luiz Antonio Munhoz da Cunha

**Affiliations:** 1 Departamento de Ortopedia Pediátrica, Hospital Pequeno Príncipe, Curitiba, PR, Brasil; 2 Departamento de Ortopedia e Traumatologia, Hospital de Clínicas da Universidade Federal do Paraná, Curitiba, PR, Brasil

**Keywords:** orthopedics, preoperative care, three-dimensional printing

## Abstract

As in many areas of knowledge, rapid prototyping technology or additive manufacturing, popularly known as three-dimensional (3D) printing, has been gaining ground in medicine in recent years, with different applications. Numerous are the benefits of this science in orthopedic surgery, by allowing the conversion of imaging tests into 3D models. Therefore, the aim of the present study is to describe a practical step-by-step for the printing of parts from patient imaging. This is a methodological study, considering preoperative computed tomography (CT) scans of patients with orthopedic deformities. Initially, the digital imaging and communications in medicine (DICOM) examination should be imported into the 3D reconstruction software of anatomical structures for the segmentation and conversion process to the stereolithography (STL) format. The next step is to import the STL file into the 3D modeling software, which allows you to work freely by manipulating the 3D mesh. The 3D models were printed additively on the GTMax3D Core A3v2 fused deposition modeling (FDM) technology printer.

## Introduction

As in many areas of knowledge, rapid prototyping technology or additive manufacturing, popularly known as three-dimensional (3D) printing, has been gaining ground in medicine in recent years, with different applications. 1 The benefits of this science in orthopedic surgery are abundant, by allowing the conversion of imaging tests into 3D models ( Table 1 ). 1 2 3 4 Among them, we mention the printing of anatomical models for a better understanding of deformities and complex fractures, the performance of safer and individualized preoperative planning, patient education, and professional training. 3 4 More recently, much has been studied regarding the printing of custom implants, bone substitutes, and biological materials. 5 It is believed that rapid prototyping can revolutionize the treatment of several orthopedic disorders. 6

**Table 1:** Applicability of Rapid Prototyping Technology in Orthopedics

Printing of anatomical models
Better understanding of diseases
Individualized preoperative planning
Custom implants
Bone substitutes
Education
Patient-specific guides
Custom orthotics

**Sources:** Wong 3 ; Ejnisman et al. 4 ; Larsen et al. 5

Studies on additive manufacturing in orthopedics, despite exposing results that favor the treatment of patients in various ways, do not explain in detail how to print the models from the imaging exams. Therefore, considering the multiple possibilities of performing protocols for 3D printing of anatomical models, the aim of the present study is to describe a practical step-by-step for the printing of parts from patient imaging.

## Description of Materials and Technique

The research project was approved by the research ethics committee of the institution, as well as the waiver of the free and informed consent form (CAAE: 48296321.8.0000.0097).

This is a methodological study, considering preoperative computed tomography (CT) scans of patients with bone deformities in the upper or the lower limbs, performed in the multislice computed tomography device of the hospital, GE Healthcare Revolution, 64 channels (GE Healthcare, Chicago, IL *,* USA).

### Exam Download and Segmentation

Initially, the digital imaging and communications in medicine (DICOM) CT scan is downloaded directly from the imaging visualization system. Then, DICOM files are imported into the InVesalius (Centro de Tecnologia da Informação Renato Archer, Brazil) version 3.1.1, a free software for reconstruction of images from CT or magnetic resonance imaging (MRI) equipment, so that the segmentation process is performed, that is, the selection of the area of interest for printing.

To perform segmentation, you must follow the following steps: (1) upload the data; (2) select the region of interest; (3) set up the 3D surface; and (4) export the data.

To import the exam, you must click on "import medical images" and select the DICOM images from the exam. Among all the sequences imported into the software, choose preferably the one with the largest number of cuts available so that the reconstruction has the best possible resolution. The smaller the thickness of the tomographic sections and the distance between them, the better the quality of the reconstruction. Once the images have been selected, you must click "import" in the lower left corner of the screen.

**Fig. 1:**
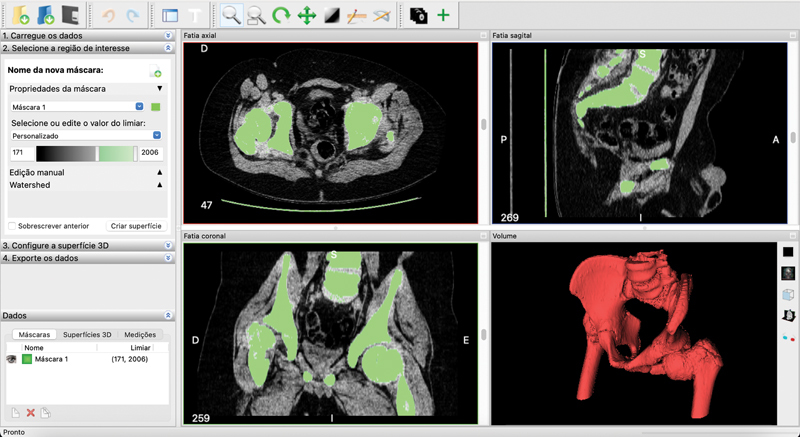
Segmentation or selection of the area of interest in multiplanar images. The bone part of the pelvis and the hips was selected and adjusted in a personalized way varying the selection threshold (in green). After that, the 3D surface was created (in red). Image obtained by the authors in the InVesalius software (Centro de Tecnologia da Informação Renato Archer, Brazil).

The second step in targeting is selecting the region of interest. The program automatically selects an exam area in multiplanar view. This selection can be customized with the brush tool or with the custom adjustment of the selection threshold. Once that is done, click "create surface." The 3D surface will appear in the lower right square of the screen ( Fig. 1 ).

Then, the created 3D surface can be configured, if necessary, in "advanced options". The fourth and final step of segmentation is data exporting. You must click "export 3D surface" and save the file in stereolithography (STL) format.

### 3D Mesh Edition

Once the segmentation is complete, the next step is to import the STL file into Meshmixer software (Autodesk Inc. **,** San Rafael, CA, United States) version 3.5.474, which allows you to work freely by manipulating the 3D mesh to later materialize the project on the printer.

**Fig. 2:**
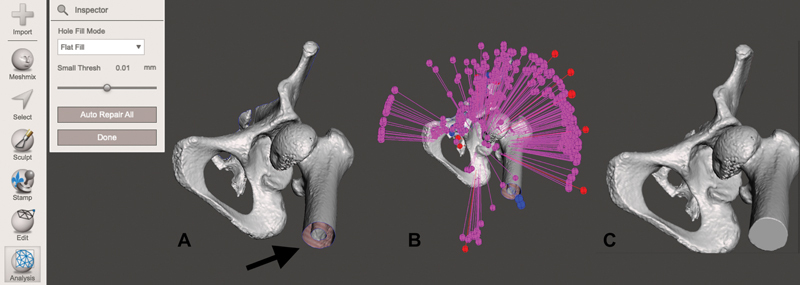
Analysis of the 3D mesh surface for zones that cannot be printed due to insufficient surface. In ( **A** ), the cross-section of the femoral dayphysis with insufficient surface (arrow) is observed. In ( **B** ), the analysis was done automatically by the inspector tool, and, in ( **C** ), the previously insufficient surface is already filled. Image obtained by the authors in Meshmixer software (Autodesk, San Rafael, CA, USA).

**Fig. 3:**
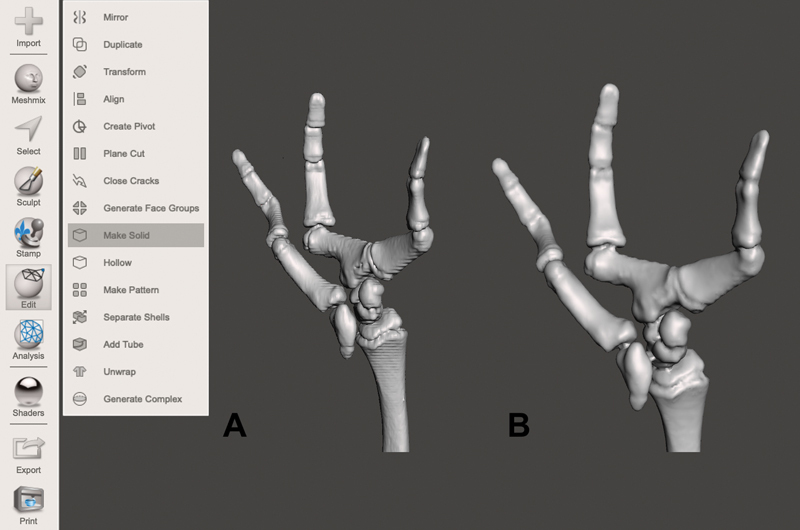
Transform the object into a solid. In ( **A** ), the selected area is observed in the segmentation step. Observe the appearance of the connectors joining the carpal bones in ( **B** ), important for the printing of a single object. Image obtained by the authors in Meshmixer software (Autodesk **,** San Rafael, CA, USA).

This program has a multitude of functions for 3D modeling. Among the main functions, we mention selecting, sculpting, mesh surface analysis ( Fig. 2 ), and transforming the object into a solid ( Fig. 3 ).

### Slicer and Printing

Once the 3D mesh editing process is complete, you must save the file in STL format and transfer it to the slicer software Cura (Ultimaker, Utrecht, Netherlands) version 4.3 to perform the model print settings. The 3D models were printed additively on the GTMax3D Core A3v2 fused deposition modeling (FDM) technology printer. The material used for printing was styrene butadiene acrylonitrile (SBA), available in filament form with a diameter of 1.75mm.

The first step is to add a printer to the software. Once this is done, the program makes the print desk available with the dimensions of the selected printer. Click "File" > "Open file" and select the edited STL file.

In this software, you must configure the print parameters. The settings depend on the printer model and on the material used. In the case of the printer and the material available in the laboratory of our institution, the following general settings are used: printing speed of 100 mm/s; 0.15 mm layer thickness; 250°C printing temperature; 10% fill density.

**Fig. 4:**
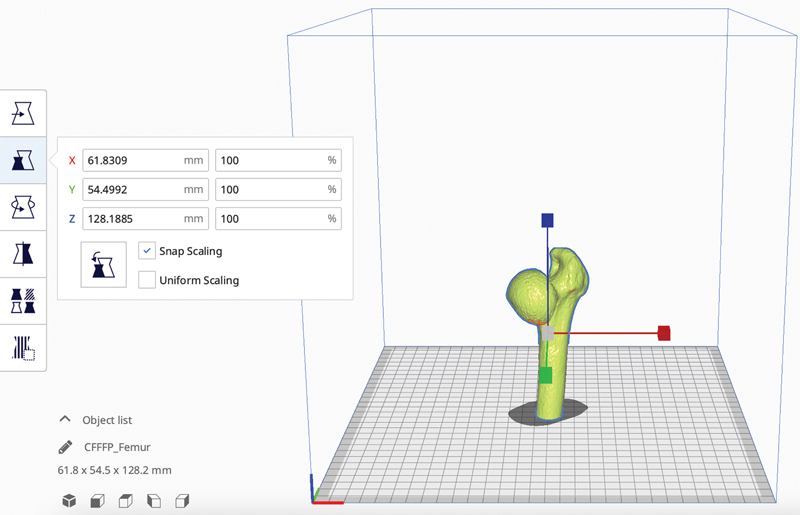
The "scale" function allows you to configure the dimensions of the object. Image obtained by the authors in Cura software (Ultimaker, Utrecht, Netherlands)

It is also important to be aware of some main functions of the Cura software, such as the scale function ( Fig. 4 ), to define the dimensions of the part, and the supports' function, so that the filament has support for printing larger structures that do not have support in the lower layers.

**Fig. 5:**
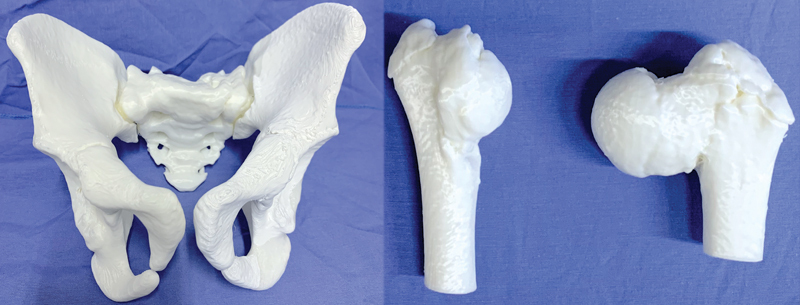
Pelvis and proximal regions of the femurs of a male patient, 11 years old, with complex bone deformities, printed by additive manufacturing technology from the preoperative computed tomography examination. Image obtained by the authors.

After defining all described settings, click "slice" at the bottom right of the screen. The software performs the slicing of the object, so that it can then be revised layer by layer. The expected time for the completion of the print according to the characteristics of the part and the defined settings is also informed. For object materialization, the G-code format is saved on a memory card to transfer it directly to the 3D printer ( Fig. 5 ).

## Final Comments

The use of full-size and haptic models is particularly interesting for education as well as for a better understanding of the disease. 7 In the study by Weidert et al., even experienced surgeons show improved classification and treatment planning with the help of 3D printed models when compared with simple CT data. 8

There are several softwares available for printing and modeling 3D parts. The authors usually use the programs described in this article because they are free and have a relatively fast learning curve. There is no conflict of interest. In this section of the article, some considerations are made to facilitate the practical understanding of readers.

In the segmentation stage, the automatic selection of the area of interest is performed by tissue density. This way, it becomes relatively easy to segment the bone, because its density is very different from those of other soft tissue structures. The skeleton segmentation process is facilitated by selecting the images in the soft tissue window and not in the bone window.

In the 3D mesh editing phase, the "sculpt" tool allows the bone surface to be smoothed for better quality printing. Although it is very useful, it is extremely important to be very careful during the smoothing of the surface of the piece, so as not to distort the anatomical particularities; therefore, the authors recommend reducing the "strength of the brush" when it is necessary to use this function.

Regarding the scale function of the slicing software ( Fig. 4 ), it offers the option to scale the model to be printed. When it is necessary to make custom implants, the printing of the model in actual size (100%) is necessary. Not all 3D printers support printing large models. In these cases, you can print in parts, as shown in Fig. 5 , where the femurs were printed separately from the pelvis.

When it comes to print settings, one should bear in mind that the better the object resolution, the more print layers are required and, consequently, the longer the printing.

The choice of the printing material should also consider its own characteristics. SBA is an oil-derived thermoplastic that has excellent mechanical resistance and good resistance at high temperatures. In general, printing models using SBA requires closed printers, such as the one used in our service. Other materials are widely available and are also easy to handle, such as lactic polyacid (LPA), a thermoplastic polymer made with lactic acid from raw materials from renewable sources. Bear in mind that print settings must also be made according to the material chosen.
