# Supplementary material for: Systematization of Steps for Printing 3D Models of Orthopedic Deformities
Source: Rev Bras Ortop (Sao Paulo). 2022 Jun 6;59(4):e626–31. doi: 10.1055/s-0042-1748816 (PMC11374395; doi:10.1055/s-0042-1748816)
Supplement: Related texts

# Research article: Sistematização das etapas para impressão de modelos 3D de deformidades ortopédicas

**Keywords:** cuidados pré-operatórios, impressão tridimensional, ortopedia

## Abstract

Assim como em diversas áreas do conhecimento, a tecnologia de prototipagem rápida ou manufatura aditiva, conhecida popularmente como impressão tridimensional (3D), vem ganhando espaço na medicina nos últimos anos, com diferentes aplicações. Inúmeros são os benefícios desta ciência na cirurgia ortopédica, ao permitir a conversão de exames de imagem em modelos tridimensionais. Diante disso, o objetivo do presente estudo é descrever um passo-a-passo prático para a impressão de peças a partir de exames de imagem de pacientes. Trata-se de um estudo metodológico, considerando exames de tomografia computadorizada (TC) pré-operatórios de pacientes portadores de deformidades ortopédicas. Inicialmente, o exame em formato digital imaging and communications in medicine (DICOM, na sigla em inglês) deve ser importado para o software de reconstrução tridimensional de estruturas anatômicas para o processo de segmentação e de conversão para o formato stereolithography (STL, na sigla em inglês). A próxima etapa é a importação do arquivo STL para o software de modelagem 3D, o qual permite trabalhar de forma livre manipulando a malha 3D. Finalizado o processo de edição, deve-se transferir o arquivo para o software fatiador para realizar as configurações de impressão e para salvar o arquivo em formato G-code para a materialização do objeto na impressora. Os modelos 3D foram impressos de forma aditiva, na impressora de tecnologia fused deposition modeling (FDM, na sigla em inglês) GTMax3D Core A3v2.

## Introdução

Assim como em diversas áreas do conhecimento, a tecnologia de prototipagem rápida ou manufatura aditiva, conhecida popularmente como impressão tridimensional (3D), vem ganhando espaço na medicina nos últimos anos, com diferentes aplicações. 1 Inúmeros são os benefícios desta ciência na cirurgia ortopédica, ao permitir a conversão de exames de imagem em modelos tridimensionais ( Tabela 1 ). 1 2 3 4 Dentre eles, cita-se a impressão de modelos anatômicos para a melhor compreensão de deformidades e de fraturas complexas, a realização de planejamentos pré-operatórios mais seguros e de forma individualizada, a educação de pacientes e o treinamento de profissionais. 3 4 Mais recentemente, muito tem se estudado a respeito de impressão de implantes personalizados, substitutos ósseos e materiais biológicos. 5 Acredita-se que a prototipagem rápida pode revolucionar o tratamento de diversas afecções ortopédicas. 6

**Tabela 1:** Aplicabilidade da tecnologia de prototipagem rápida na ortopedia

Impressão de modelos anatômicos
Melhor compreensão das doenças
Planejamentos pré-operatórios individualizados
Implantes personalizados sob medida
Substitutos ósseos
Educação
Guias específicos do paciente
Órteses sob medida

**Fontes:** Wong 3 ; Ejnisman et al. 4 ; Larsen et al. 5

Os estudos sobre manufatura aditiva na ortopedia, apesar de exporem resultados que favoreçam o tratamento dos pacientes de diversas formas, não detalham, de maneira pormenorizada, como realizar a impressão dos modelos a partir dos exames de imagem. Diante disso, considerando as múltiplas possibilidades de se realizar protocolos para impressão 3D de modelos anatômicos, o objetivo do presente estudo é descrever um passo-a-passo prático para a impressão de peças a partir de exames de imagem de pacientes.

## Descrição dos materiais e técnica

O projeto de pesquisa foi aprovado pelo comitê de ética em pesquisa da instituição, assim como a dispensa do termo de consentimento livre e esclarecido (CAAE: 48296321.8.0000.0097).

Trata-se de um estudo metodológico, considerando exames de tomografia computadorizada (TC)pré-operatórios de pacientes portadores de deformidades ósseas nos membros superiores ou inferiores, realizados no aparelho de tomografia computadorizada multislice do hospital, GE Healthcare Revolution, 64 canais (GE Healthcare, Chicago, IL *,* Estados Unidos *)* .

## Download do exame e segmentação

Inicialmente, é feito o download do exame de TC no formato digital imaging and communications in medicine (DICOM, na sigla em inglês) diretamente do sistema de visualização de imagens. Em seguida, os arquivos DICOM são importados para o software InVesalius (Centro de Tecnologia da Informação Renato Archer, Brasil) versão 3.1.1, programa livre para reconstrução de imagens provenientes de equipamentos de TC ou ressonância magnética (RM), para que seja realizado o processo de segmentação, ou seja, a seleção da área de interesse para impressão.

Para realizar a segmentação, é necessário seguir as seguintes etapas: (1) carregar os dados; (2) selecionar a região de interesse; (3) configurar a superfície 3D; e (4) exportar os dados.

Para importar o exame, deve-se clicar em "importar imagens médicas" e selecionar as imagens DICOM do exame. Dentre todas as sequências importadas para o software, escolher, preferencialmente, a com a maior quantidade de cortes disponíveis para que a reconstrução tenha a melhor resolução possível. Quanto menor for a espessura dos cortes tomográficos e a distância entre eles, melhor será a qualidade da reconstrução. Uma vez selecionadas as imagens, deve-se clicar em "importar" no canto inferior esquerdo da tela.

O segundo passo da segmentação é a seleção da região de interesse. O programa seleciona automaticamente uma área do exame na visualização multiplanar. Esta seleção pode ser customizada com a ferramenta de pincel ou com o ajuste personalizado do limiar de seleção. Feito isso, clique em "criar superfície". A superfície 3D aparecerá no quadrado inferior direito da tela ( Fig. 1 ).

**Fig. 1:** Segmentação ou seleção da área de interesse nas imagens multiplanares. A parte óssea da pelve e dos quadris foi selecionada e ajustada de maneira personalizada variando o limiar de seleção (em verde). Depois, foi criada a superfície 3D (em vermelho). Imagem obtida pelos autores no *software* InVesalius (Centro de Tecnologia da Informação Renato Archer, Brasil).

Em seguida, a superfície 3D criada poderá ser configurada, se necessário, em "opções avançadas". O quarto e último passo da segmentação é a exportação dos dados. Deve-se clicar em "exportar superfície 3D" e salvar o arquivo em formato stereolithography (STL, na sigla em inglês).

## Edição da malha 3D

Uma vez finalizada a segmentação, a etapa seguinte é a importação do arquivo STL para o software Meshmixer (Autodesk Inc **.,** San Rafael, CA, Estados Unidos) versão 3.5.474, que permite trabalhar de forma livre manipulando a malha 3D para, posteriormente, materializar o projeto na impressora.

Este programa possui uma infinidade de funções para modelagem 3D. Dentre as funções principais, cita-se a de selecionar, a de esculpir, a de análise da superfície da malha ( Fig. 2 ) e a de transformar o objeto em um sólido ( Fig. 3 ).

**Fig. 2:** Análise da superfície da malha 3D à procura de zonas que não podem ser impressas por motivo de superfície insuficiente. Em A, observa-se o corte transversal da diáfise do fêmur com superfície insuficiente (seta). Em (B), a análise foi feita automaticamente pela ferramenta *inspector* e, em (C), a superfície previamente insuficiente já está preenchida. Imagem obtida pelos autores no *software* Meshmixer (Autodesk Inc ***.*** **,** San Rafael, CA, Estados Unidos).

**Fig. 3:** Transformar o objeto em um sólido. Em ( **A** ), observa-se a área selecionada na etapa de segmentação. Observar o aparecimento dos conectores unindo os ossos do carpo em ( **B** ), importante para a impressão de um único objeto. Imagem obtida pelos autores no software Meshmixer (Autodesk Inc ***.*** **,** San Rafael, CA, Estados Unidos).

## Fatiador e impressão

Finalizado o processo de edição da malha 3D, deve-se salvar o arquivo em formato STL e transferi-lo para o software fatiador Cura (Ultimaker, Utrecht, Holanda), versão 4.3, para realizar as configurações de impressão do modelo. Os modelos 3D foram impressos de forma aditiva, na impressora de tecnologia fused deposition modeling (FDM, na sigla em inglês) GTMax3D Core A3v2. O material utilizado para impressão foi o acrilonitrila butadieno estireno (ABS), disponibilizado em forma de filamento com diâmetro de 1,75mm.

O primeiro passo é adicionar uma impressora no software. Feito isso, o programa disponibiliza a mesa de impressão já com as dimensões da impressora selecionada. Clique em "Arquivo" > "Abrir arquivo" e selecione o arquivo STL editado.

Neste programa, é necessário configurar os parâmetros de impressão. As configurações dependem do modelo da impressora e do material utilizado. No caso da impressora e do material disponível no laboratório da nossa instituição, utilizam-se as seguintes configurações gerais: velocidade de impressão de 100 mm/s; espessura da camada de 0,15 mm; temperatura de impressão de 250°C; densidade de preenchimento de 10%.

É importante também que se tenha conhecimento de algumas funções principais do software Cura, como a função de escala ( Fig. 4 ), para definir as dimensões da peça, e a função de suportes, para que o filamento tenha apoio para impressão de estruturas maiores que não possuem apoio nas camadas inferiores.

**Fig. 4:** A função "escala" permite configurar as dimensões do objeto. Imagem obtida pelos autores no software Cura (Ultimaker, Utrecht, Holanda).

Após realizar todas as configurações descritas, clique em "fatiar" na parte inferior direita da tela. O programa realiza o fatiamento do objeto, de modo que este pode, então, ser revisado camada a camada. Também é informado o tempo previsto para a conclusão da impressão de acordo com as características da peça e com as configurações definidas. Para a materialização do objeto, salva-se o arquivo no formato G-code em um cartão de memória para transferi-lo diretamente para a impressora 3D ( Fig. 5 ).

**Fig. 5:** Pelve e regiões proximais dos fêmures de paciente masculino, 11 anos, portador de deformidades ósseas complexas, impressas por meio da tecnologia de manufatura aditiva a partir do exame de tomografia computadorizada pré-operatória. Imagem obtida pelos autores.

## Comentários finais

O uso de modelos em tamanho real e hápticos é particularmente interessante não somente para a educação, mas também para o melhor entendimento da doença. 7 No estudo de Weidert et al., mesmo cirurgiões experientes mostram classificação e planejamento de tratamento aprimorados com a ajuda de modelos impressos em 3D quando comparados com os dados simples de TC. 8

Existem diversos *softwares* disponíveis para impressão e modelagem de peças 3D. Os autores costumam utilizar os programas descritos no presente artigo por serem gratuitos e com uma curva de aprendizado relativamente rápida. Não há conflito de interesses. Nesta seção do artigo, algumas considerações são feitas para facilitar o entendimento prático dos leitores.

Na etapa de segmentação, a seleção automática da área de interesse é realizada pela densidade dos tecidos. Desta forma, torna-se relativamente fácil segmentar o osso, porque a densidade é muito diferente das demais estruturas de partes moles. O processo de segmentação do esqueleto é facilitado ao selecionar as imagens em janela de partes moles e não em janela óssea.

Na fase de edição da malha 3D, a ferramenta "esculpir" permite que a superfície do osso seja suavizada para uma impressão de melhor qualidade. Apesar de muito útil, é de extrema importância que se tome muito cuidado durante a suavização da superfície da peça para não distorcer as particularidades anatômicas; por isso, os autores recomendam reduzir a "força do pincel" quando for necessário o emprego desta função.

Em relação à função de escala do software fatiador ( Fig. 4 ), ela oferece a opção de dimensionar o modelo a ser impresso. Quando é preciso confeccionar implantes sob medida, a impressão do modelo no tamanho real (100%) se faz necessária. Nem todas as impressoras 3D suportam a impressão de modelos grandes. Nestes casos, pode-se imprimir a peça em partes, como mostrado na Fig. 5 , na qual os fêmures foram impressos separadamente da pelve.

No que diz respeito às configurações de impressão, deve-se ter em mente que quanto melhor a resolução do objeto, mais camadas de impressão são necessárias e, consequentemente, mais tempo de impressão.

A escolha do material de impressão também deve levar em consideração suas características próprias. O ABS é um termoplástico derivado do petróleo que possui ótima resistência mecânica e boa resistência a altas temperaturas. De modo geral, a impressão de modelos utilizando o ABS necessita de impressoras fechadas, como a utilizada no nosso serviço. Outros materiais estão amplamente disponíveis e também são de fácil manuseio, como o poliácido láctico (PLA), um polímero termoplástico feito com ácido lático a partir de matérias-primas de fontes renováveis. Lembre-se de que as configurações de impressão também devem ser feitas de acordo com o material escolhido.
